# A legend forever – Dr. Brij Bhushan Joshi

**Published:** 2009

**Authors:** Ram Prabhoo

**Affiliations:** *Vice President, Indian Orthopaedic Association and Consultant Orthopaedic surgeon, Mukund Hospital Marol, Andheri Kurla Road Andheri East, Mumbai - 400059, India* E-mail: aprabhoo@hotmail.com


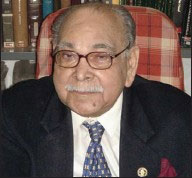


Dr. Brij Bhushan Joshi (22.08.1928 - 08.06.2009)

Dr. Brij Bhushan Joshi is indeed a legend. He has been acclaimed as a “Pioneer in Hand Surgery”. His genius and innovativeness put him in a class of his own. His single minded dedication to his patients kept him up many nights but this perseverance led him to find solutions where none existed.

He started his medical career at the prestigious KEM Medical College in Lahore. After partition, he shifted to Grant Medical College and Sir J. J. Group of hospitals in Bombay and qualified as the first M. S. in Orthopedics from the University of Bombay in 1950. He started off as the youngest Chief of Surgery at Irwin Hospital, Delhi at the age of 27.

His affinity for hand surgery came at the M.G.M. Hospital in Mumbai where workers with complex hand injuries, caused by a variety of industrial machines, came in large numbers. He tirelessly researched all available knowledge and filled gaps by thinking up a simple but functionally effective solution that enabled him to send his patient back to work. His operative skill was almost magical as his knowledge of anatomy enabled him to ‘see’ through the skin and pin point a structure by meticulous tissue handling. He devised new sensory flaps for restoring ‘sight’ to a blind patient's fingertips. He discovered early that even the best surgery must be supplemented with effective splints and exercises. In a small room adjacent to his 30 bedded ward he started a splint workshop as he found that effective splints were not available. He devised economic and effective splints made from wire, aluminum strips, plastic tubes, felt, buttons and rubber bands, often bought in bulk from the junk market!

Mr. Guy Pulvertaft, the “Father of Modern Hand Surgery” was amazed by the variety and the novelty in Dr. Joshi's work and he persuaded Dr. Joshi to present a paper on an international platform in 1974. Always shy of public speaking, Dr. Joshi needed to boost his confidence by practicing his speech in front of his friends a few times before delivering it to a large audience! The impact of his work sent signals across the globe as hand surgeons accepted Dr. Joshi's ideas wholeheartedly. When Dr. Joshi devised the ‘percutaneous’, 3 point ‘K’ wire fixation of fracture of the shaft of the proximal phalanx, he cemented his reputation worldwide.

After retirement from M.G.M. Hospital, Dr. Joshi turned his focus to mini external fixator for the hand popularly known now as JESS. Once again, he took an everyday object and transformed a part of an electrical plug into a link joint. He took this breakthrough further by developing several effective frames for minimally invasive treatment of many hand problems. He then extended this logic to feet, especially clubfeet, and made a device for simultaneous correction of the deformity in three planes in preambulatory age children. The result was supple, fully corrected feet by the time the child started walking! He organized workshops, seminars and lectures to popularize his devices but it is a tribute to his generosity that he never patented them!

I had the privilege to enjoy a very special relation with him for the last three decades. He was not only a philosopher, mentor and guide to me, but just like a father he taught me to think and serve the poor and needy, especially the rural population of India. His knowledge of history, appreciation of art and subtle sense of humor was known to a very few and close associates of his. He also initiated me to do “VIDYADAN” to all budding orthopedic surgeons all over India, which I have been committed to for the last 15 years.

His legacy continues as all our students hold seminars, conduct workshops, deliver lectures dissipating his brilliant innovations far and wide for generations to come.

He was extremely fulfilled when his work was published in the form of three knowledge packed books in June, 2007. His family, colleagues, students, patients and co-workers will always remember him fondly and miss him sorely.

